# Next-generation Bruton tyrosine kinase inhibitors and degraders in the treatment of B-cell malignancies: advances and challenges

**DOI:** 10.1007/s00277-025-06515-7

**Published:** 2025-07-28

**Authors:** Yao Wang, Yaping Zhang, Jiaqi Liu, Yongning Jiang, Jianyong Li, Wenyu Shi

**Affiliations:** 1 Department of Oncology, Affiliated Hospital of Nantong University, Nantong, China; 2 Department of Hematology, Affiliated Hospital 6 of Nantong University, Yancheng Third People’s Hospital, Yancheng, China; 3 Department of Hematology, Affiliated Hospital of Nantong University, Nantong, China; 4 Nantong University, Nantong, China; 5 Department of Hematology, Jiangsu Province Hospital, First Affiliated Hospital of Nanjing Medical University, Nanjing, China

**Keywords:** Bruton tyrosine kinase, Non-covalent BTK inhibitors, BTK PROTACs, B-cell malignancies, Efficacy, Safety

## Abstract

Bruton tyrosine kinase (BTK), a key component of B-cell receptor signaling, is crucial for the development of B-cell malignancies. Covalent BTK inhibitors (cBTKis), such as ibrutinib, have demonstrated remarkable efficacy, but their curative potential is limited by acquired resistance. Next-generation BTK inhibitors, including non-covalent BTK inhibitors and BTK Proteolysis-targeting chimeras, offer new options for patients who have developed resistance to cBTKis. Some of these inhibitors have shown favorable efficacy and safety profiles, leading to Food and Drug Administration approval. This review summarizes the current landscape of BTK inhibitors, focusing on the evolution from cBTKis to next-generation inhibitors in terms of clinical efficacy and challenges, such as resistance mechanisms and off-target effects. We conclude with an outlook on future research and clinical applications.

## Background

B-cell malignancies, including B-cell leukemias and lymphomas, are among the most common hematological malignancies [1]. Bruton tyrosine kinase (BTK), a key kinase in the downstream signaling pathway of the B-cell receptor (BCR), is essential for signal transduction in normal B cells and has been implicated in the pathogenesis of B-cell malignancies [2, 3]. BTK activity supports the survival and proliferation of malignant B cells, making BTK inhibition a key therapeutic approach in B-cell malignancies [4]. The Food and Drug Administration (FDA) approval of the first covalent BTK inhibitor (cBTKi), ibrutinib, for mantle cell lymphoma (MCL), chronic lymphocytic leukemia (CLL), and Waldenström’s macroglobulinemia (WM) marked the beginning of a chemotherapy-free era in the management of B-cell malignancies [5–7]. Since then, more selective BTK inhibitors, such as acalabrutinib, zanubrutinib, and orelabrutinib, have been developed for the treatment of these malignancies [3, 8–10]. Despite the remarkable clinical efficacy of these BTK inhibitors, reports of drug resistance and adverse effects are common. Evidence suggests that nongenetic adaptation mechanisms to BTK inhibition exist in CLL during early therapy [11]. However, the genetic mechanisms of BTKi resistance are more prevalent. The inhibitors target the BTK protein at the cysteine-481 (C481) residue, and studies have shown that C481 mutations, particularly a cysteine-to-serine substitution (C481S) [12], can disrupt cBTKi binding, as a key underlying mechanism for acquired resistance [13–15]. Moreover, the inhibition of kinases that contain cysteine residues homologous to C481 in BTK can result in undesirable off-target effects [16–19]. To overcome these limitations, next-generation BTK inhibitors have been developed. Non-covalent BTK inhibitors, also known as reversible inhibitors, bind to BTK through non-covalent interactions rather targeting C481, offering an option for patients who are resistant or intolerant to cBTKis [20]. Proteolysis-targeting chimeras (PROTACs), which recruit an E3 ligase to degrade both wild-type and C481S-mutant BTK through ubiquitination, represent another therapeutic option [21, 22].

### Mechanism of action of BTK inhibitors

**Fig. 1:**
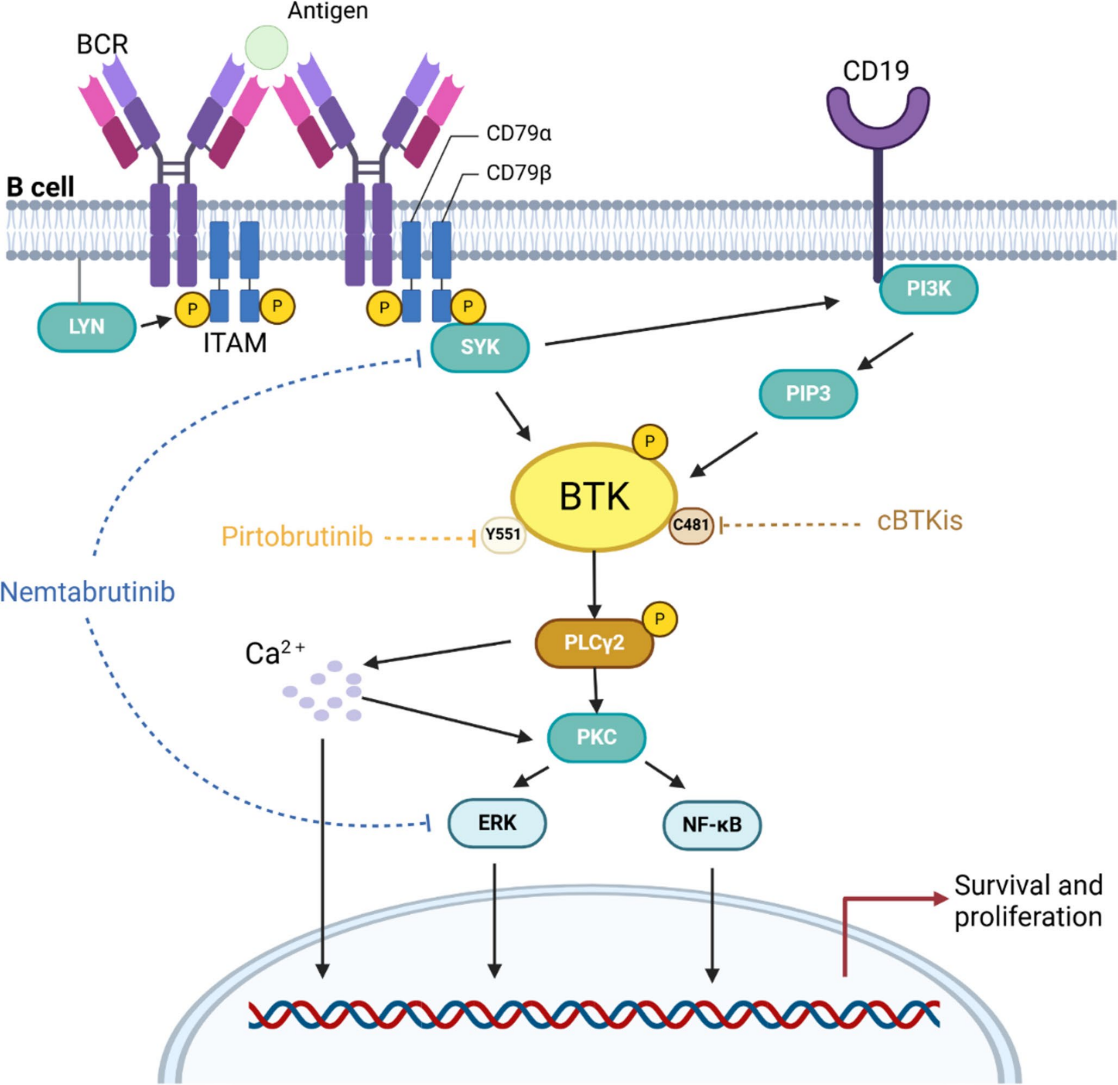
BCR signaling pathway. Upon antigenic stimulation, BCR undergoes accumulation to recruit CD79α/β combined with LYN kinase on lipid rafts, and subsequently recruits and phosphorylates SYK kinase, followed by ITAMs.The activated ITAMs induce activation of the downstream BTK and phosphoinositide 3-kinase (PI3K). BTK, as a key effector of PI3K, then activates the downstream PLCγ2, further amplifying the BCR signals. Further downstream responses include calcium (Ca^2+^) mobilization as well as activation of protein kinase C (PKC) and the MAPK/ERK and NF-κB pathways, contributing to the survival and proliferation of B cells. Aberrant activation of the BCR signaling pathway supports the development of malignant neoplastic cells and the ways in which cBTKis and some non-covalent BTK inhibitors inhibit BCR signaling are also shown

**Fig. 2:**
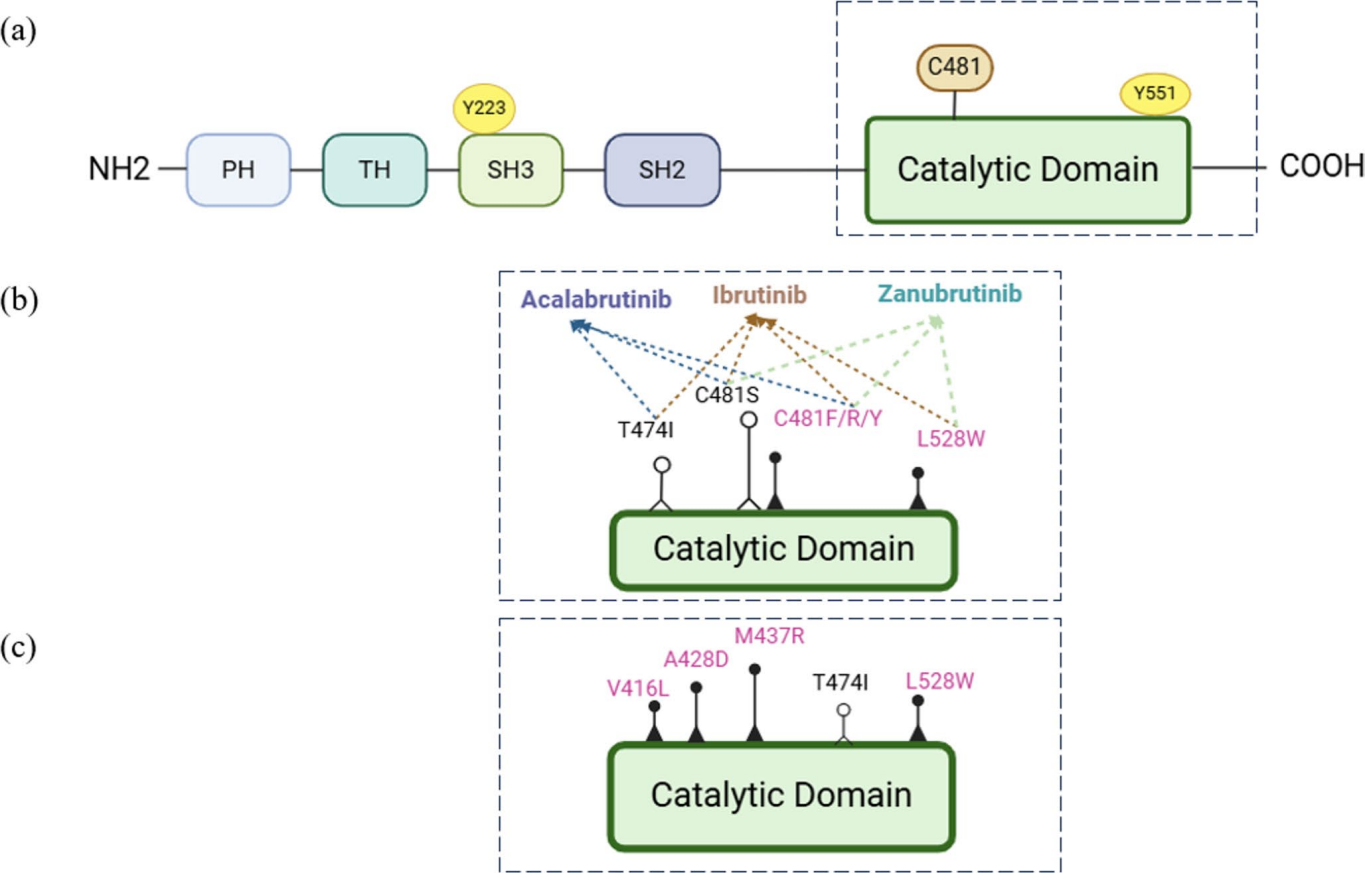
(**a**) Structural diagram of BTK. The BTK protein consists of five domains from the N-terminus to the C-terminus: a pleckstrin homology (PH) domain, a proline-rich TEC homology (TH) domain, two SRC homology (SH) domains (SH2 and SH3), and a catalytic domain. Y223 in the SH3 domain and Y551 in the catalytic domain are the two critical tyrosine phosphorylation sites in BTK. When the BCR signaling pathway is activated, SYK enhances the catalytic activity by phosphorylation of Y551 and promotes the subsequent self-phosphorylation of Y223. Consequently, BTK inhibitors that bind to the catalytic domain block the subsequent Y223 self-phosphorylation. (**b**)Resistance mutations of some cBTKis.(**c**) Resistance mutations of the pirtobrutinib

Activation of the BCR signaling pathway is critical for B-cell proliferation and survival. Antigenic stimulation activates BCR in normal B cells, leading to the phosphorylation of intracellular immunoreceptor tyrosine-based activation motifs (ITAMs) in CD79α/β via SYK kinase. The phosphorylated ITAMs activate BTK and the downstream phospholipase Cγ2 (PLCγ2), thereby amplifying BCR signaling and activating the MAPK/ERK and nuclear factor (NF)-κB pathways [23] **(**Fig. 1**)**. This signaling cascade has been co-opted by malignant B cells. Evidence has shown that chronic active BCR signaling leads to constitutive activation in activated B-cell-type diffuse large B-cell lymphoma (ABC-DLBCL) [24], and BCR signaling appears to be a major driver of proliferation in CLL [25], MCL [26], and follicular lymphoma (FL) [27]. BTK, a pivotal upstream node in the BCR network, is activated by phosphorylation of Y551 in the catalytic domain of SYK, promoting self-phosphorylation of Y223 in the SRC homology 3 (SH3) domain [23, 28] **(**Fig. 2a**)**. Thus, targeted inhibition of BTK is a validated therapeutic strategy for B-cell malignancies, as evidenced by current clinical applications.

Ibrutinib selectively binds to C481 in the catalytic domain of BTK, thereby inhibiting Y223 autophosphorylation, which suppresses BCR signaling and ultimately leads to death of malignant B cells [29]. Ibrutinib was the first BTK inhibitor approved by the FDA, and it has achieved remarkable results in the treatment of various B-cell malignancies, both as a monotherapy and in combination regimens [5–7, 30–33]. However, off-target effects have limited its use, with hematological toxicities being the most common and cardiovascular adverse events being the most concerning. A long-term follow-up study indicated that ibrutinib had a cumulative discontinuation rate of 19% for pneumonia, anemia, atrial fibrillation, diarrhea, subdural hematoma, and thrombocytopenia, partly attributed to its off-target effects [34]. Recently developed cBTKis have demonstrated higher selectivity and reduced toxicity. In a phase 3 trial, acalabrutinib exhibited non-inferior progression-free survival (PFS) compared with ibrutinib and some adverse events were less common, especially fewer cardiovascular adverse events [35]. Zanubrutinib demonstrated improved efficacy and safety outcomes over ibrutinib owing to its higher activity and bioavailability, particularly in patients with chronic lymphocytic leukemia/small lymphocytic lymphoma (CLL/SLL) and MCL, as shown in the ALPINE study (NCT03734016) [36]. Phase 2 studies of orelabrutinib in B-cell malignancies indicated improved safety arising from its high selectivity [37, 38]. Tirabrutinib showed superior selectivity and extended efficacy in patients with relapsed or refractory (R/R) primary central nervous system lymphoma [39].

However, continuous therapy with cBTKis has been complicated by the emergence of resistance. These cBTKis exert their effects by covalently binding to C481 [40]. Studies have indicated that most BTK mutations affect the C481 site targeted by cBTKis, leading to its substitution with a different amino acid (C481S) [14, 41–43], thus resulting in resistance to these drugs. Other substitutions at the same site, such as the conversion of C481 to phenylalanine (C481F), tyrosine (C481Y), or arginine (C481R), along with some less common mutations in PLCγ2, can also arise, conferring resistance to cBTKis [41–44] **(**Fig. 2b**)**.

### Non-covalent BTK inhibitors

Non-covalent BTK inhibitors offer an alternative approach to targeting of the BTK pathway by functioning without binding to C481, effectively inhibiting both the wild-type and C481S-mutant forms of BTK. Several agents have been investigated at the preclinical stage, with some showing promising efficacy and manageable safety profiles in clinical trials [13] **(**Table 1**)**.

**Table 1:**
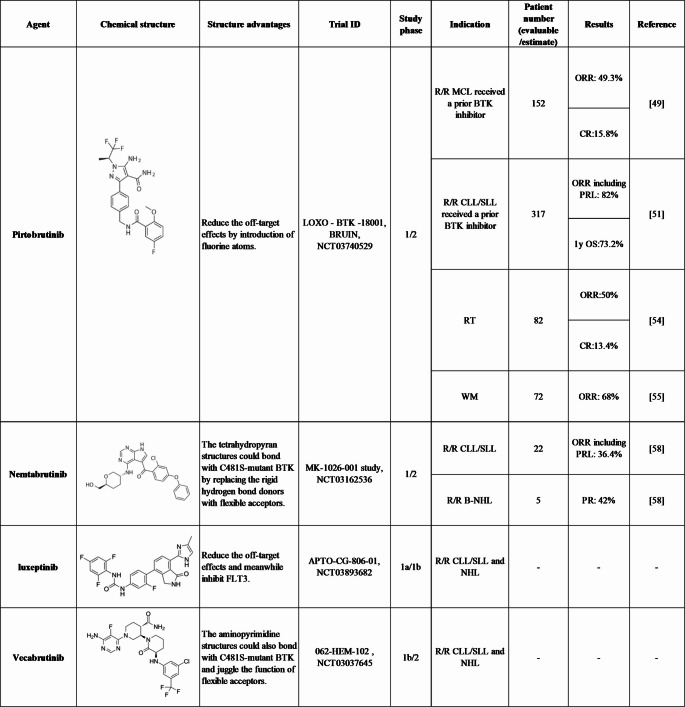
Chemical structures and clinical trials of non-covalent BTK inhibitors with available studies.

#### Pirtobrutinib (LOXO-305)

Pirtobrutinib is the first non-covalent BTK inhibitor approved by the FDA for patients with R/R MCL after two or more lines of systemic therapy, including previous BTK inhibitors [45]. It has also been approved for adult patients with CLL/SLL who have received at least two prior lines of therapy, including a BTK inhibitor and a B-cell lymphoma 2 (BCL2) inhibitor [46]. On 28 February 2025, the Committee for Medicinal Products for Human Use (CHMP) of the European Medicines Agency (EMA) adopted a positive opinion for approval of pirtobrutinib for the treatment of patients with R/R CLL who have been previously treated with a BTK inhibitor [47].

Preclinical studies have demonstrated that pirtobrutinib inhibits both wild-type and C481S-mutant BTK with low-nanomolar potency. Rather than directly interacting with C481, pirtobrutinib achieves its effects through extensive hydrogen bonding to other BTK residues and water molecules near the ATP-binding site, and acts similarly for both wild-type and C481S-mutant BTK [48]. Pirtobrutinib also prevents the phosphorylation of Y551 in the BTK activation loop, a key process for the recruitment of hematopoietic cell kinase (HCK) to kinase-dead BTK mutant proteins. This mechanism may explain how these mutant proteins continue to signal despite possessing an inactive kinase site [49]. The results of these preclinical experiments lay the foundation for subsequent clinical applications of pirtobrutinib.

Pirtobrutinib was initially approved by the FDA for the treatment of adult patients with R/R MCL based on an open-label, single-arm, international, phase 1/2 study, the BRUIN trial (NCT03740529). The study enrolled 104 evaluable patients, including 90 who had received prior cBTKi therapy and 14 who were cBTKi-naïve [50]. Among the primary efficacy cohort (*n* = 90), the objective response rate ORR was 57.8% (95% confidence interval [CI], 46.9 to 68.1), with 20.0% achieving complete response (CR) and 37.8% achieving partial response (PR). Patients with prior BTKi treatment had an ORR of 52.7%. After a median follow-up of 12 months, the median duration of response (DOR) was 21.6 months (95% CI, 7.5 to not reached), and patients with prior BTKi therapy had a median DOR of 14.8 months (95% CI, 5.55 to not reached). Regarding the treatment-emergent adverse events (TEAEs), fatigue (29.9%), diarrhea (21.3%), and dyspnea (16.5%) were common. TEAEs of Grade 3 or higher were hemorrhage (3.7%) and atrial fibrillation/flutter (1.2%). Only 3% of patients discontinued treatment because of a TEAE [50]. More patients with R/R MCL who received prior cBTKi therapy had been recruited and the update data of the BRUIN MCL at the data cut-off of 5 May 2023 revealed thatt he efficacy results showed that the cBTKi-pretreated patients (*n* = 152) had an ORR of 49.3% (95% CI, 41.1 to 57.6), with 15.8% achieving CR and 33.6% achieving PR, while the cBTKi-naïve patients (*n* = 14) had an ORR of 85.7% (95% CI, 57.2 to 98.2) [51]. The ORR of patients who discontinued a prior cBTKi because of progressive disease (PD) was 43.0%. In the safety data, the most common TEAE of Grade 3 or higher was neutropenia/neutrophil count decreased (13.3%), while hemorrhage (2.4%) and fibrillation/flutter (3.6%) remained infrequent. Overall, 8 patients (5%) had TEAEs leading to dose reductions and 5 patients (3%) had TEAEs leading to discontinuation [51].

In a preclinical study for CLL, pirtobrutinib was proven to continuously inhibit the BCR pathway in both wild-type and C481S-mutant BTK CLL cells [52], providing a rationale for the treatment of patients refractory to cBTKis. The BRUIN trial explored the efficacy and safety of pirtobrutinib in a total of 317 patients with CLL/SLL [46]. Among the enrolled patients, 274 had received prior treatment with at least one cBTKi, and approximately 100 patients had received a BCL2 inhibitor such as venetoclax. Among the patients with prior cBTKi treatment, the ORR was 73.3% (95% CI, 67.3 to 78.7), with 1.6% achieving CR, 0.4% achieving nodular PR, and 71.3% achieving PR. The percentage increased to 82.2% (95% CI, 76.8 to 86.7) when partial response with lymphocytosis (PRL) was included. For patients who had previously received both a BTK inhibitor and a BCL2 inhibitor, the ORR was 70.0% (95% CI, 60.0 to 78.8), and the percentage increased to 79.0% (95% CI, 69.7 to 86.5) when PRL was included. Common TEAEs included infections (71.0%), bleeding (42.6%), and neutropenia (32.5%). Compared with those with other BTK inhibitors, the rates of atrial fibrillation (3.8%), major hemorrhage (2.2%), and hypertension (14.2%) were lower. Only 9 patients (2.8%) discontinued pirtobrutinib because of a TEAE. Updated data were disclosed at the 2023 American Society of Hematology (ASH) annual meeting. Enrolled patients with prior BTK inhibitor treatment had increased to 282 at the cut-off of 5 May 2023. The patients showed an ORR including PRL of 82% (95% CI, 76.5 to 85.9) regardless of previous therapy, age, or mutation status. After a median follow-up of 27.5 months, the median PFS was 19.4 months (95% CI, 16.6 to 22.1) [53]. Frequent TEAEs were fatigue (36.9%), neutropenia (34.4%), diarrhea (28.4%), cough (27.3%), and contusion (26.2%). The phase 1b portion of the BRUIN trial further investigated the comparative therapeutic efficacies of pirtobrutinib plus venetoclax (PV) and PV plus rituximab (PVR) as a 2-year fixed-duration therapy [54]. The efficacy results showed an ORR of 96% (95% CI, 79.6 to 99.9), with 40% achieving CR (PV 28%; PVR 12%) and 56% achieving PR (PV 28%; PVR 28%) across the two arms. The most common Grade 3 or higher TEAE was neutropenia/neutrophil count decreased (PV 46.7%; PVR 60.0%). TEAEs led to dose reductions in 3 patients (1 with PV and 2 with PVR), and treatment discontinuation in only 2 patients with PVR. The findings indicated that pirtobrutinib has potential for combined therapy with other molecular targeted agents in CLL [46].

Approximately 10% of CLL patients experience an aggressive histological transformation called the Richter transformation (RT) that frequently leads to large B-cell lymphoma [55]. These patients are currently treated with regimens derived from the DLBCL treatment paradigm or participate in small-scale clinical trials because no standard treatment is available. Data from the phase 1/2 BRUIN study presented at the 2023 ASH annual meeting showed that pirtobrutinib was a potential option for these patients [56]. For all 82 patients enrolled, the ORR was 50.0% (95% CI, 38.7 to 61.3), with 13.4% achieving CR and 36.6% achieving PR. Frequent TEAEs were neutropenia/neutrophil count decreased (29.3%), fatigue (24.4%), and diarrhea, dyspnea, thrombocytopenia, and pyrexia (18.3% each). Only 3 patients had TEAEs leading to dose reductions, while no patients discontinued treatment [56].

The results for the WM cohort in the BRUIN study were also reported. WM, a rare indolent lymphoma, was defined as a malignant lymphoplasmacytic clone residing in the bone marrow and producing a monoclonal IgM paraprotein. Among 72 response-evaluable WM patients, the major response rate was 68% (95% CI, 56 to 79), with 24% achieving very good partial response (VGPR) and 44% achieving PR. For patients who had received prior cBTKi therapy, the major response rate was 64% (95% CI, 50 to 76), with 24% achieving VGPR and 40% achieving PR. In the safety data, TEAEs of Grade 3 or higher included hypertension (3%), hemorrhage (2%), and atrial fibrillation/flutter (1%), and 15 (2%) patients discontinued treatment. The results indicated that pirtobrutinib was well tolerated as a therapy for WM patients [57].

Taken together, pirtobrutinib provides a new opportunity for treatment of B-cell malignancies, including some rare hematological disorders. Furthermore, the efficacy and safety of pirtobrutinib in patients with R/R FL or marginal zone lymphoma (MZL) are currently being examined. Several ongoing phase 3 clinical trials, such as BRUIN CLL-322 (NCT04965493) and BRUIN MCL-321 (NCT04662255), are ongoing in more homogeneous populations of patients with B-cell malignancies. The results are eagerly awaited.

#### Nemtabrutinib(MK-1026, ARQ 531)

Nemtabrutinib, a non-covalent BTK inhibitor currently under investigation, is an oral drug designed to inhibit oncogenic BCR signaling by targeting both wild-type and mutant BTK proteins. Preclinical studies revealed that nemtabrutinib inhibited kinases upstream of the BTK signaling pathway, inducing cytotoxicity in CLL cells regardless of the presence of C481S or PLCγ2 mutations. PLCγ2 mutations represent another mechanism of resistance to cBTKis [58]. Nemtabrutinib was also able to inhibit distal targets including MEK1 in the ERK signaling pathway, which was identified as an effective target in diffuse large B-cell lymphomas [59].

The first-in-human phase 1 study of nemtabrutinib (MK-1026-001 study; NCT03162536) evaluated its efficacy in patients with R/R CLL, B-cell NHL, or WM, all of whom had undergone at least two prior treatments [60]. Among the 47 enrolled patients (29 with CLL, 17 with NHL, and 1 with WM), 8 CLL patients achieved at least PRL, with 1 achieving CR, 6 achieving PR, and 1 achieving PRL. In the phase 2 trial, the patients achieved an ORR of 75% [60]. In terms of safety, 81% experienced at least one TEAE of Grade 3 or higher, with the most common being neutropenia (23.4%), febrile neutropenia (14.9%), and infectious pneumonia (14.9%). Most adverse events were Grade 1 or 2 and were generally tolerable, indicating a manageable safety profile. Currently, several phase 3 global, randomized, open-label clinical studies, including BELLWAVE-011 (NCT06136559) evaluating newly diagnosed CLL and SLL patients, and BELLWAVE-008 (NCT05624554) focusing on previously untreated CLL and SLL patients without TP53 aberrations, are underway and hold promise [61].

#### Other non‑covalent BTK inhibitors

In addition to the agents with fast-track development above, there are several non-covalent BTK inhibitors under investigation. A previous study on luxeptinib (CG-806), a non-covalent pan-BTK/pan-FLT3 inhibitor, demonstrated favorable safety and tolerability [62]. Further recruitment of patients with R/R CLL/SLL and NHL is ongoing for a clinical study (NCT03893682). Preclinical studies on another non-covalent BTK inhibitor, vecabrutinib, revealed ITK-inhibitory properties similar to ibrutinib and demonstrated equal efficacy for both C481S-mutant and wild-type BTK proteins [63, 64]. Furthermore, in vivo mouse experiments showed that vecabrutinib reduced the tumor burden and significantly improved survival in the murine Eµ-TCL1 adoptive transfer model, and had a synergistic effect with venetoclax [63]. A phase 1b/2 trial (NCT03037645) is currently investigating the efficacy and safety in humans, and its results are awaited. Some other non-covalent BTK inhibitors, such as HMPL-760 (CTR20213134) and HBW-3210 (CTR20233167), are undergoing clinical trials for R/R B-cell non-Hodgkin lymphomas(B-NHL). In the meantime, several non-covalent BTK inhibitors have shown potential for the treatment of autoimmune diseases and chronic kidney disease, including fenebrutinib (GDC-0853), SN1011, and EVER001 (XNW1011). Further clinal trials are ongoing or in the planning stage.

Non-covalent BTK inhibitors have shown broad application prospects with favorable safety and tolerability to overcome the emergence of cBTKi resistance. Potential applications of non-covalent BTK inhibitors in B-cell malignancies are worthy of further attention.

### BTK PROTACs

**Fig. 3:**
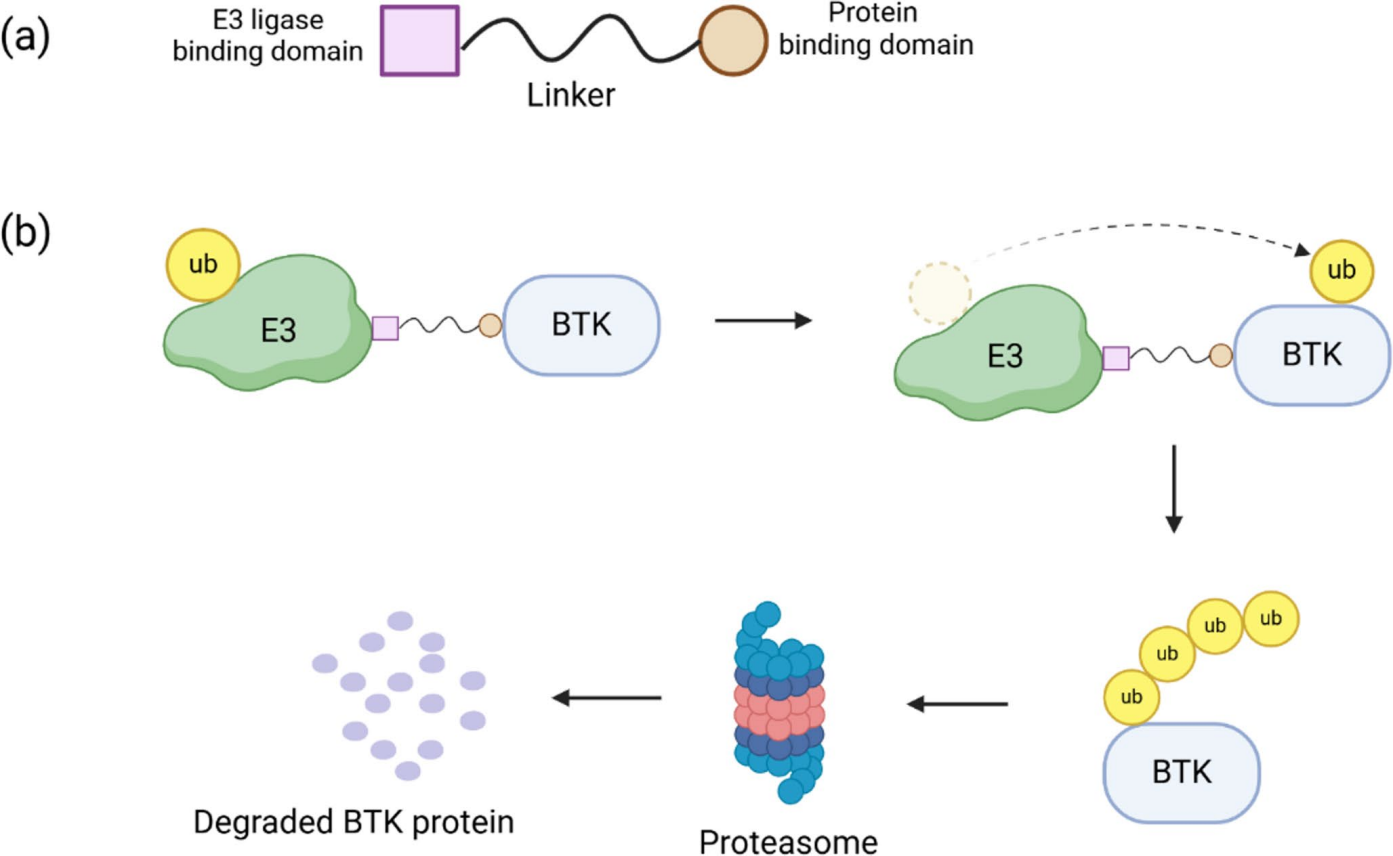
(**a**)Structural diagram of a BTK PROTAC. The BTK PROTAC is composed of a ligand for the target protein, a ligand for the E3 ubiquitin ligase, and a linker chain. (**b**) Mechanism of action of the PROTAC. Upon binding to the BTK protein, the PROTAC recruit E3 ubiquitin ligase, leading to ubiquitination of the target protein, followed by its proteasomal degradation

Independently of C481 mutations, new resistance-associated mutations have emerged in patients undergoing clinical trials of non-covalent BTK inhibitors [52]. A recent study evaluated 9 patients with R/R CLL who underwent pirtobrutinib therapy using single-cell DNA sequencing, and identified several BTK mutations (V416L, A428D, M437R, T474I, and L528W) that conferred resistance to both non-covalent BTK inhibitors and some cBTKis [65] **(**Fig. 2c**)**. Furthermore, mutations in PLCγ2, a signaling molecule and downstream substrate of BTK, were found in all 9 patients, rendering their malignant cells less dependent on BTK protein [43, 65]. Another study revealed recurrent BTK mutations in two distinct enzymatic groups: kinase-proficient mutations (T474I and C481S), and mutations reducing BTK enzymatic function (M437R, V416L, C481F, C481R, C481Y, and L528W) [66] **(**Fig. 2**)**. These mutated proteins acquired new functions, promoting abnormal B-cell proliferation and contributing to disease relapse by acting as bridging scaffold proteins that directly bind to downstream components of the BCR pathway, such as HCK and ILK (hematopoietic cell kinases) [66]. PROTACs, a new class of non-covalent BTK inhibitors, are composed of three components: a ligand for the target protein, a ligand for the E3 ubiquitin ligase, and a linker chain **(**Fig. 3a**)**. This structure allows PROTACs to recruit the E3 ubiquitin ligase, leading to ubiquitination of the target protein, followed by its proteasomal degradation **(**Fig. 3b**)**. These new drugs may address some of the challenges associated with traditional small molecule-based inhibitors. The current research findings for BTK PROTACs are summarized in Table 2.

**Table 2:**
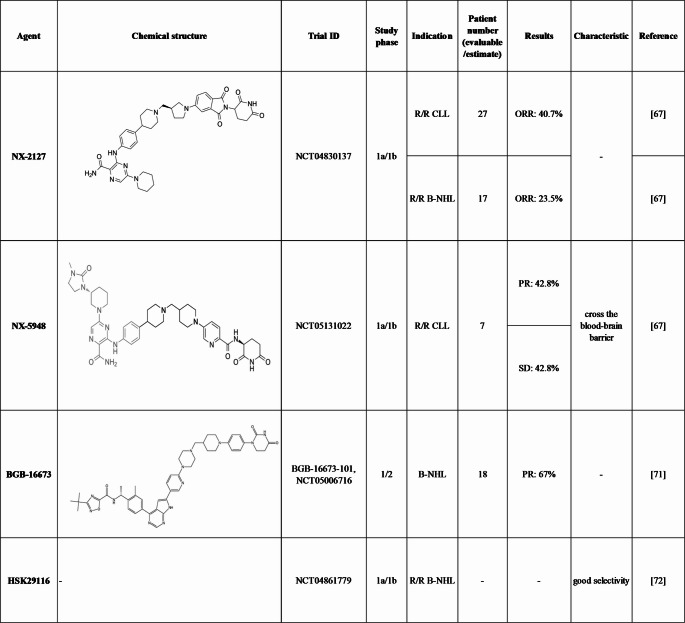
Chemical structures and clinical trials of BTK PROTACs

#### NX-2127

NX-2127, a first-in-class heterobifunctional and orally bioavailable BTK degrader, can potently and selectively target BTK, IKZF1, and IKZF3 for proteasomal degradation. The agent exhibited in vivo degradation across species, and demonstrated efficacy in preclinical oncology models [67]. On this basis, it was evaluated in a phase 1a/1b trial for treatment of patients with B-cell malignancies (NCT04830137). In the clinical trial, all enrolled patients had previously received a BTKi, and 76.5% had also been treated with venetoclax. Mutations were observed in BTK (C481, 29%; L528, 29%; T474, 14%; V416, 7%) and BCL2 (14%). The ORR was 33%, and the incidence of TEAEs of Grade 3 or higher was 58%, as of 16 June 2022 [68]. At the 2023 ASH annual meeting, 11 of the 27 evaluable CLL patients experienced PR with an ORR of 40.7%. Furthermore, 1 MCL patient and 1 DLBCL patient achieved CR that lasted for > 1 year. In addition, 1 MZL patient and 1 FL patient had PR. Regarding safety, 38.9% patients had serious TEAEs including dose-related neutropenia, hypertension, and anemia, of which 14.8% were related to NX-2127 treatment [69]. Furthermore, NX-2127 was able to bind to all mutant forms of BTK examined, including those with resistance mutations to non-covalent BTK inhibitors. Clinical application of NX-2127 in CLL achieved > 80% degradation of BTK in patients, and the TEAEs were tolerable and manageable [66]. Although the clinical study had to be suspended for manufacturing process improvements, the improved agents are worth waiting for, based on their potential for their clinical application.

#### NX-5948

NX-5948, another oral small-molecule degrader of BTK, was first disclosed at the 2023 ASH annual meeting. Research has suggested that it can degrade both wild-type and mutant BTK proteins. It has been shown to cross the blood–brain barrier and degrade BTK in microglia and brain-resident lymphoma cells in preclinical studies [70]. Data from the dose escalation stage of a phase 1a/1b clinical trial evaluating daily oral dosing of NX-5948 in patients with R/R B-cell malignancies were revealed. Among 7 CLL patients who received doses of 50–200 mg, 6 patients showed clinical benefits, with 3 patients achieving PR and 1 patient experiencing PR for > 9 months. Three patients showed stable disease (SD) and 2 patients were still receiving treatment [69]. According to the safety data, NX-5948 was well-tolerated across doses of 50–450 mg, with no serious adverse events observed and no patients who discontinued treatment. NX-5948 is currently being evaluated in a phase 1 clinical trial (NCT05131022) for patients with R/R B-cell malignancies. More clinical data are eagerly anticipated.

#### BGB-16673

BGB-16673 is another orally available BTK degrader designed to target both wild-type and multiple mutant forms of BTK. It exhibited potent anti-proliferative activity in cellular experiments and showed stronger inhibition of BTK and PLCγ2 phosphorylation than ibrutinib and pirtobrutinib [71, 72]. In mouse xenograft models of wild-type and C481S-mutant BTK lymphoma, BGB-16673 was associated with better survival and less splenic tumor invasion than cBTKis and non-covalent BTK inhibitors [72]. Results for BGB-16673 in a phase 1 trial, BGB-16673-101 (NCT05006716), were disclosed at the 2023 ASH annual meeting. The trial included 26 patients with R/R CLL, WM, MCL, MZL, non-germinal center B-cell DLBCL, FL, or RT [73]. Overall, 77% patients remained on the therapy, with 4 patients discontinuing treatment because of PD and 2 patients withdrawing from the trial. Among the 18 response-evaluable patients, 67% responded and 23% achieved PR, including patients who had previously received a cBTKi (*n* = 10) and a non-covalent BTK inhibitor (*n* = 2). Moreover, all responders remained in response and exhibited sustained reductions in the BTK protein levels in their peripheral blood or tumor tissue, indicating favorable long-term efficacy. TEAEs were reported in 16 patients, 46.2% with TEAEs of Grade 3 or higher, and contusion, pyrexia, neutropenia/neutrophil count decreased, and lipase increased were common [73]. BGB-16673 has demonstrated a tolerable safety profile and good clinical responses thus far, and more long-term clinical data are awaited for further evaluation.

#### Other BTK PROTACs

Some BTK PROTACs have exhibited significant antitumor effects in preclinical studies. HSK29116, a small BTK PROTAC molecule, inhibited cell proliferation without measurable loss of other BTK-family kinase activities, thereby limiting off-target toxicities [74]. At the 2023 American Association for Cancer Research annual meeting, the methods for a phase 1 study (NCT04861779) on HSK29116 in patients with R/R B-cell malignancies were reported. This phase 1 trial intended to include dose escalation to identify dose-limiting toxicities and to establish the maximum tolerated dose recommended for the phase 1b dose expansion. The safety, pharmacokinetics, investigator-assessed ORR, duration of response, time to response, and PFS would be assessed [74].

HZ-Q1070 is another promising candidate for clinical application. It exhibited excellent pharmacokinetic properties, BTK degradation activity, and robust tumor growth inhibition in cell experiments and a mouse tumor model. HZ-Q1070 was also shown to avoid degradation of Aiolos and Ikaros, which are important for NK cell development, and in turn protect the body against pathogens and cancers [75]. The clinical efficacy and safety of this BTK PROTAC warrant further evaluation.

Taken together, BTK PROTACs promote the degradation of both wild-type and mutant BTK proteins and are more flexible and efficient for inhibition of B cell growth, thus improving the duration and effectiveness of BTK-based treatment. This strategy is potentially important for improving the prognosis and survival in patients with B-cell malignancies. We look forward to more data becoming available for evaluation of the efficacy and safety of BTK PROTACs.

## Conclusions

BTK is a crucial component of BCR signaling and plays a significant role in the development and progression of B-NHL. Previous studies on cBTKis, such as ibrutinib, zanubrutinib, and acalabrutinib, have shown favorable efficacy and safety for patients with B-NHL, but their application is limited by drug resistance and off-target toxicities. Non-covalent BTK inhibitors, including BTK PROTACs, allow patients to regain the benefits of targeting the BTK pathway, and have demonstrated efficacy and safety in preclinical studies. Several non-covalent BTK inhibitors are under investigation and hold promise for long-term outcomes. Thus, re-establishing therapeutic strategies for BTK inhibition is a potential option for patients in whom previous cBTKi therapy has been interrupted for reasons such as PD or toxicity. BTK PROTACs are also poised to become the first-line therapy for patients with B-cell malignancies because of their mechanism of action.

## Data Availability

No datasets were generated or analysed during the current study.

## Abbreviations

BTK: Bruton tyrosine kinase
cBTKis: Covalent BTK inhibitors
BCR: B-cell receptor
FDA: The Food and Drug Administration
MCL: Mantle cell lymphoma
CLL: Chronic lymphocytic leukemia
WM: Waldenström’s macroglobulinemia
C481: Cysteine-481
C481S: C481 mutation of a cysteine-to-serine substitution
PROTACs: Proteolysis-targeting chimeras
ITAMs: Immunoreceptor tyrosine-based activation motifs
PLCγ2: Phospholipase Cγ2
ABC-DLBCL: Activated B-cell-type diffuse large B-cell lymphoma
FL: Follicular lymphoma
SH3: SRC homology 3
PFS: Progression-free survival
CLL/SLL: Chronic lymphocytic leukemia/small lymphocytic lymphoma
R/R: relapsed or refractory
C481F: C481 mutation of a cysteine-to-phenylalanine substitution
C481Y: C481 mutation of a cysteine-to-tyrosine substitution
C481R: C481 mutation of a cysteine-to-arginine substitution
BCL2: B-cell lymphoma 2
HCK: Hematopoietic cell kinase
DOR: Duration of response
TEAEs: Treatment-emergent adverse events
PRL: Partial response with lymphocytosis
ASH: American Society of Hematology
PV: Pirtobrutinib plus venetoclax
PVR: PV plus rituximab
RT: Richter transformation
VGPR: Very good partial response
MZL: Marginal zone lymphoma
ILK: Hematopoietic cell kinases
SD: Stable disease
PI3K: Phosphoinositide 3-kinase
Ca^2+^: Calcium
PKC: Protein kinase C
PH: pleckstrin homology
TH: TEC homology
